# Evaluation and prevention and control measures of urban public transport exposure risk under the influence of COVID-19—Taking Wuhan as an example

**DOI:** 10.1371/journal.pone.0267878

**Published:** 2022-06-06

**Authors:** Huan Lu, Hongcheng Gan

**Affiliations:** 1 Department of Transportation System Engineering, University of Shanghai for Science and Technology, Shanghai, P.R. China; 2 Center for Supernetworks Research, University of Shanghai for Science and Technology, Shanghai, P.R. China; Monash University, AUSTRALIA

## Abstract

**Background:**

Since December 2019, COVID-19 began to spread throughout the world for nearly two years. During the epidemic, the travel intensity of most urban residents has dropped significantly, and they can only complete inflexible travel such as "home to designated hospital" and "home to supermarket" and some special commuting trips. While ensuring basic travel of residents under major public health emergency, there is also a problem of high risk of infection caused by exposure of the population to the public transport network. For the discipline of urban transport, how to use planning methods to promote public health and reduce the potential spread of diseases has become a common problem faced by the government, academia and industry.

**Method:**

Based on the mobility perspective of travel agents, the spatial analysis methods such as topological model of bus network structure, centrality model of public transport network and nuclear density analysis are used to obtain the exposure risk and spatial distribution characteristics of public transport from two aspects of bus stops and epidemic sites.

**Results:**

The overall spatial exposure risk of Wuhan city presents an obvious "multi center circle" structure at the level of bus stops. The high and relatively high risk stops are mainly transport hubs, shopping malls and other sites, accounting for 35.63%. The medium and low-risk stops are mainly the villages and communities outside the core areas of each administrative region, accounting for 64.37%. On the other hand, at the scale of epidemic sites, the coverage covers 4018 bus stops in Wuhan, accounting for 36.5% of all bus stops, and 169 bus lines, accounting for 39.9% of all routes. High risk epidemic sites are mainly concentrated in the core areas within the jurisdiction of Wuhan City, and in the direction of urban outer circle diffusion, they are mainly distributed in the low and medium risk epidemic sites. According to the difference of the risk level of public transport exposure, the hierarchical public transport control measures are formulated.

**Discussion:**

This paper proposes differentiated prevention and control countermeasures according to the difference of risk levels, and provides theoretical basis and decision-making reference for urban traffic management departments in emergency management and formulation of prevention and control countermeasures.

## 1. Introduction

At the end of 2019, COVID-19 (Corona Virus Disease 2019) suddenly began to break out and spread in Wuhan, China [1]. Since then, the COVID-19 has become a global pandemic, cases have surged in more than 200 countries all over the world including the United States, South Korea, Iran, and Italy. As a major public health emergency, COVID-19 has become a significant issue of concern to the whole society due to the long duration, large number of affected people, and large social impact. Urban public health problems have once again become the focus of attention in many disciplines such as medicine, planning, geography, management, psychology, and environmental science. As the COVID-19 is highly contagious, many countries around the world have initiated emergency responses to major public health emergencies, and residents’ awareness of their own safety protection has gradually increased. Most obviously, the travel intensity of most urban residents has dropped significantly, and they can only complete inflexible travel such as "home to designated hospital" and "home to supermarket" and some special commuting trips. In order to complete these trips, urban bus is still an open urban commuter in many cities. However, railway transport (metro, inter-city train) usually stop operation. Because people believe that the subway and urban railway are a more closed riding environment than urban bus, and there are more people taking a single ride, so people are more likely to be infected with the virus. Therefore, this paper studies the relationship between urban bus network and epidemic situation, which can reflect the relationship between public transport and covid-19 to a certain extent. While ensuring basic travel of residents under major public health emergency, there is also a problem of high risk of infection caused by exposure of the population to the public transport network. For the discipline of urban transport, how to use planning methods to promote public health and reduce the potential spread of diseases has become a common problem faced by the government, academia and industry.

The existing research on emergency management of major public emergency originated from the field of management, involving the theory, system and evaluation of emergency management [2, 3]. In recent years, the traffic system plays a key role in the occurrence of major emergency, so many scholars have focused on the model, method and management strategy of urban traffic emergency management. The related research mainly focuses on the transport emergency management in the face of natural disasters and traffic collisions [4–6]. In response to major public health emergency, targeted traffic emergency management strategies are indispensable, and exposure risk is an important measurement method [7]. The research on exposure risk mainly focuses on geography, health science, disaster science and traffic safety. For example, Kousa quantified the exposure risk based on the concentration of air pollutants and the spatial distribution of population, and realized the high-precision spatial simulation of exposure risk [8]; Pan revealed that the IQ of children exposed to heavy metal environmental pollution in industrial areas was significantly lower than that in nonindustrial areas [9]. With the in-depth study of exposure risk [10], it is gradually found that ignoring the temporal and spatial changes of risk factors and the exposure in the process of people’s travel activities will lead to the misjudgment of risk level, also the research on the exposure degree in the process of travel has attracted more and more attention. Due to the different scenarios, different modes of transport and different crowd behavior patterns, the measurement methods of exposure risk are not unified, and the theoretical research on urban transport exposure risk is still relatively rare.

Previous studies have shown that urban traffic planning can affect human health by influencing people’s travel patterns. The corresponding action path is to reduce the relative exposure (volume and time) in traffic environment through road traffic design, so as to reduce the risk of traffic collisions [11, 12]. In order to explore the relationship and mechanism between urban spatial elements and health effects, researchers usually collect spatial data, and establish buffer, irregular polygon and regular polygon as analysis units based on certain spatial data processing rules. Although the theoretical level has proved the significant correlation and action path between urban spatial elements and non-communicable diseases [13–16], whether these factors also have the same impact on infectious diseases remains to be further verified. Due to the difficulty in obtaining fine-grained and small-scale infectious disease data, the current research on COVID-19 mainly focuses on the analysis of epidemiological characteristics based on Statistics [17, 18], the origin and evolution of virus based on gene comparative analysis [19, 20], and epidemic response From the three aspects of emergency management and control [21, 22], empirical research on external factors influencing COVID-19 communication risk from the spatial perspective is still lacking. COVID-19 is characterized by high risk of infection and long incubation period. Residents’ unconscious interactive travel and traffic exposure will increase the risk of cross infection [23]. Through the management of residents’ public transportation travel behavior, the risk of public transportation exposure to residents can be reduced, thereby reducing the risk of infection. In the long run, this is an important theoretical basis for reducing the potential risk of the epidemic and enhancing the prevention of the epidemic among residents.

In summary, existing studies have provided some insights into urban public transport emergency management under major public health emergency. However, due to the lack of basic research, the overall transport emergency and management work under major public health emergency is relatively passive. Especially in the special scenario of COVID-19 transmission, urban traffic emergency management and the public transport exposure risk assessment under residents’ inelastic travel demand is particularly important. Therefore, in light of the traditional research cannot accurately identify public transport exposure risk under major public health emergency, we construct public transport exposure risk assessment method, and analyze the impact of residents’ daily trips on public transport exposure risk systematically. We obtain public transport exposure risk evaluation indicators and their spatial distribution characteristics, and discover public bus stops and epidemic sites with high exposure risks. This paper takes Wuhan, the place where COVID-19 occurs in China as a case study. The conclusion obtained in this study provides a theoretical basis for the emergency management and control of urban public transport under the control of the spread of the epidemic.

## 2. Methodology

In this paper, the spatial analysis methods such as topological model of bus network structure, centrality model of public transport network and nuclear density analysis are used to obtain the exposure risk and spatial distribution characteristics of public transport from two aspects of bus stops and epidemic sites. Considering that the spread of epidemic diseases in urban public transport system is a dynamic process driven by network, it is difficult to distinguish it from external factors due to the multi-scale characteristics of human mobility network and its own heterogeneity. At the same time, when the public transport network is faced with the challenge of major public health events, the travel demand of residents will change under the strong control of policies. However, the conventional public transport network evaluation method does not take into account the interactive exposure risk of travel agents in the whole public transport system in a specific time and space. Therefore, based on the mobility perspective of travel agents, this paper constructs a topology model of public transport network structure, and introduces the centrality of public transport network to represent the public transport exposure risk. In other words, the greater the degree of freedom of travelers in the public transport network and the intensity of external contact, the more vulnerable they are to be exposed to the infection risk of crowd interaction.

### 2.1 Construction of topological model of public transport network structure

In order to make the public transport network more in line with people’s travel habits and characteristics, this paper proposes the following assumptions to construct the topological model based on the ‘Space-L’ spatial topology mapping rules:

1. Most of the urban bus lines are bidirectional, and the stops setting and passenger flow in the two directions are basically symmetrical. In order to focus on the travelers’ travel path in the public transport network and ignore the differences in the running direction and frequency of the bus lines, the urban public transport network is regarded as an undirected non weighted network.
2. The bus line above the line direction shall prevail, ignoring the difference caused by the same name of individual stop but different location, and the same name bus stop is regarded as a node.
3. Considering that the actual bus line represents the moving path, it is considered that there are connected edges between two adjacent nodes on a random line.

### 2.2 Construction of public transport network centrality model

Transport network centrality is an effective means to measure accessibility, and it plays an important role in the theoretical and practical research of public transport planning and urban geography. Based on the constructed urban bus network topological model, the bus network centrality is used to construct a public transport exposure risk measurement model, which measures the public transport exposure risk from two aspects: accessibility and connectivity. Accessibility refers to the convenience of taking the bus to the surroundings and other areas. When a major health emergency occurs in a city, a site with higher accessibility is at a key position in the bus network. In other words, the site and its surrounding areas bear more risks. This paper uses degree centrality, betweenness centrality, and proximity centrality as indicators to characterize this feature. Site connectivity refers to the connection efficiency between sites, and is characterized by a clustering coefficient. A higher clustering coefficient means that the connection between stops and stops in a local area is closer. The greater the traffic flow into the stop, the easier the load of the stop will spread to the surrounding area.

(1) Degree centrality.

The degree centrality indicates the probability that the stop has direct contact with other stops in the bus network. The greater the degree centrality of the stops, the higher the strength of the connection with other stops. The site location is at a key node in the network, and travelers are more likely to spread in different directions. The degree centrality can be calculated as follows

$$D_{i}=\frac{1}{d-1}n_{i}$$ (1)

Where, *D*_*i*_ is the degree centrality of the bus stop *i*; *d* is the number of all stops in the bus network; *n*_*i*_ is the total number of stops directly connected to the node *i*.

(2) Betweenness centrality

The betweenness centrality is a measurement index based on the shortest distance, which represents the number of shortest paths passing through the shortest path of all stops in the bus network. It can reflect the hub function and influence of the bus stops as a medium in the entire network. The greater the betweenness centrality is, the more time the bus travel is used. The betweenness centrality can be calculated as follows

$$B_{i}=(\frac{1}{\left(P-1)(P-2\right)}\sum_{t=1;j=1;t\neq j\neq i}^{P}\frac{m_{tj}(i)}{m_{tj}})$$ (2)

Where, *B*_*i*_ is the betweenness centrality of the stop *i*; *P* is the number of public transport network stops; *m*_*tj*_ is the number of shortest paths between the stop *t* and *j*; *m*_*tj*_(*i*) is the number of shortest paths that pass through the stop *i* in the shortest path between the stop *t* and *j*.

(3) Proximity centrality

The proximity centrality indicates the proximity a stop is to all other stops in the urban public transport network. It is measured by the reciprocal of the sum of the shortest distances from a stop to all the other stops, and represents the relative accessibility of the stop in the transport network. The greater the centrality values, the less the average cost it spends to any other node in the network, and the more it tends to the central position in the network. The proximity centrality can be calculated as follows

$$C_{i}={\left(\frac{1}{n‐1}\sum_{j=1,i\neq j}^{n}k_{ij}\right)}^{‐1}$$ (3)

Where, *C*_*i*_ is the proximity centrality of the bus stop *i*; *k*_*ij*_ is the shortest distance from the stop *i* to the stop *j*.

(4) Clustering coefficient

The clustering coefficient represents the degree of clustering of stops in the bus network, reflecting the transfer efficiency between a bus stop and other stops. The higher the stop clustering coefficient values, the higher the degree of connection between the stops and the fewer transfers. During one bus travel, this type of stop is easy to attract passengers to form a gathering. The clustering coefficient can be calculated as follows

$$CC_{i}=\frac{2T_{i}}{Q_{i}(Q_{i}+1)}$$ (4)

Where, *CC*_*i*_ is the clustering coefficient of the stop *i*; *Q*_*i*_ is the number of stops directly connected to the stop *i*; *T*_*i*_ is the number of edges between the stop *Q*_*i*_.

Based on the related relationship on the four indicators of degree centrality, betweenness centrality, proximity centrality and clustering coefficient, and the characteristics that the higher the degree of clustering of people in the spread of the epidemic, the greater the risk of infection, it is believed that betweenness centrality and clustering coefficient are the most important indicators [24, 25]. It can reflect the agglomeration and intermediary effect of the public transport network. The independent variable factor has the greatest impact on public transport exposure risk [26]. Therefore, the weight values of the four independent variable factors are determined as: 0.2, 0.3, 0.2, 0.3, and the centrality model of the public transport network can be calculated as follows

$$M_{i}=0.2D_{i}+0.3B_{i}+0.2C_{i}+0.3CC_{i}$$ (5)

Where, *M*_*i*_ is the centrality of the bus stop *i*.

### 2.3 Urban public transport exposure risk measurement method

(1) Exposure risk of bus stops

This paper calls the complex network analysis package Network X written in Python language, establishes the bus topological relationship based on the assumptions of network construction, and calculate the four indicators of degree centrality, betweenness centrality, proximity centrality and clustering coefficient and normalize them in sequence. The public transport network centrality model is used to measure the exposure risk of public transport stops. The classification of risk level is based on a clustering method commonly used in ArcGIS software: Natural Breaks method (Jenks). Considering that the impact of facilities on their service range continues to attenuate with the increase of distance, the ArcGIS kernel density analysis tool is used for bus stops and performs spatial interpolation and visual analysis to identify the spatial distribution characteristics of exposure risk of public transport stops. The kernel density calculation function can be calculated as follows

$$g(a)=\sum_{i=1}^{b}\frac{1}{l^{2}}w(\frac{a-d_{s}}{l})$$ (6)

Where, *g*(*a*) is the kernel density calculation function at the spatial location *a*; *l* is the distance attenuation threshold; *b* is the number of bus stops whose distance from the location *a* is less than or equal to *l*; *w* is the spatial weight function; *d*_s_ is the location *s* of the core key element.

(2) Exposure risk of epidemic site

Epidemic site refers to site where confirmed patients have stayed, most of which are residential communities with epidemics, except for a few supermarkets, hotels, industrial areas, etc. Taking into account the range of residents’ travel activities and the service range of urban public transport bus stops, a buffer zone is delineated with an epidemic site as the center and a radius of 500 meters. This paper develops the average of the exposure risks of public transport stops covered in the buffer zone to characterize the exposure risk of the epidemic site after it is connected to the entire public transport network. The exposure risk of epidemic sites can be calculated as follows

$$E_{q}=\sum_{i=1}^{z}M_{i}/z$$ (7)

Where, *E*_*q*_ is the exposure risk of the epidemic site *q*; *z* is number of the bus stops in the 500m buffer zone of the epidemic site *q*

## 3. Data collection

### 3.1 Study area

Wuhan is the capital of Hubei Province, one of China’s megacities, the only sub-provincial city in six provinces in central China. Wuhan is also a central city in central China approved by the State Council, and an important industrial base, science and education base and comprehensive transport hub in China. At the end of 2019, Wuhan has 13 districts with a total area of 8569.15 square kilometers, a built-up area of 812.39 square kilometers, and a permanent population of 11.212 million.

Wuhan is one of the first 15 pilot cities in China to be awarded the national demonstration project of "Transport City". Wuhan has always adhered to the concept of "Public Transport Leading Urban Development", adhered to the development strategy of "Transport Priority", guided by people’s livelihood needs, and made every effort to promote the demonstration project of "Transport City". Wuhan strives to create urban public transport that is compatible with the proposed national central city system. It performs well in terms of 500-meter stop coverage, track connection, and public transport openness. According to relevant data, the 500-meter stop coverage of Wuhan’s bus stops ranks eighth among nearly 300 prefecture level cities in China, and the bus network density ranks tenth. However, in the face of the major public health emergency COVID-19 challenge, public transport exposure risk to local residents have a great impact on their social life and physical and mental health. Therefore, this paper uses Wuhan city as the study case area to explore urban public transport exposure risk under major public health emergency.

### 3.2 Data preprocessing

**Fig 1:**
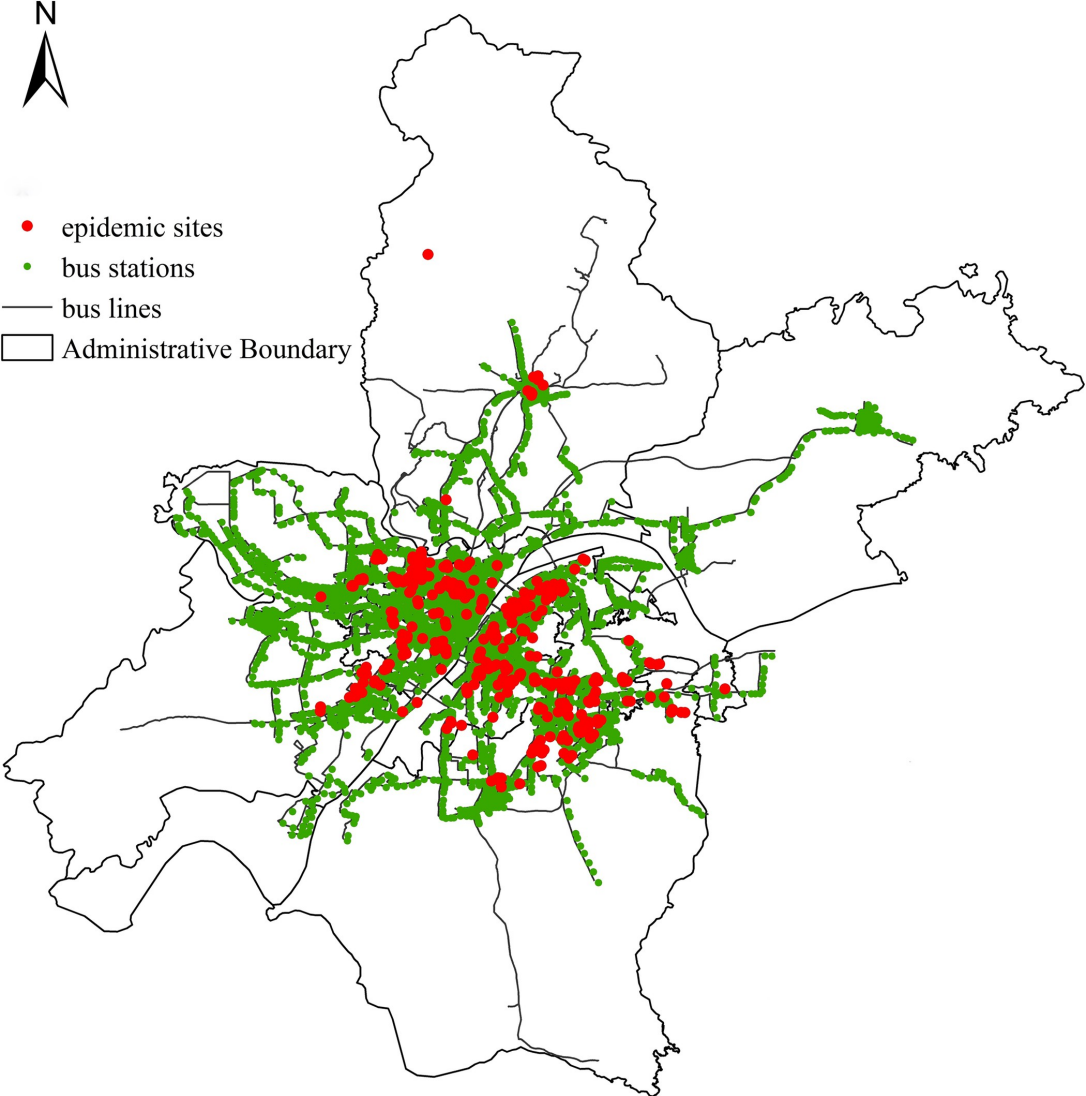
Schematic diagram of Wuhan bus network and epidemic sites.

This paper obtains the administrative area data, bus line network data, and epidemic information data of the study area through web crawler technology. Among them, basic geographic data such as urban administrative boundaries and roads come from the open source data platform OpenStreetMap (OSM). The acquisition of bus line network data is based on the Wuhan bus route table provided by the Wuhan municipal transport bureau, and the programming language Python 3.7 is used to write web crawlers, through the AutoNavi Open Platform API interface (https://lbs.amap.com). At the end of May 2020, 423 urban public bus lines (including ordinary buses, community buses, tourist dedicated lines, airport buses, etc.) in Wuhan were collected. The data mainly includes the name of the bus line, the departure time of the first and last departures, and the direction of the bus. After data cleaning, 11,009 stops are obtained. Based on the official epidemic information released by the Wuhan Health Commission, information about sites where patients diagnosed with “COVID-19” have stayed from January 30 to February 15, 2020 (called epidemic sites, including the name and time of the site) have accumulated a total of 434 items. In order to obtain the accurate location coordinates of epidemic sites, the geographic coding and reverse geocoding tools in AutoNavi Open Platform API interface were used to obtain and verify the longitude and latitude information that can reflect the center of gravity or main entrance point of the surrounding public transport network. Schematic diagram of Wuhan bus network and epidemic sites are as shown in Fig 1.

## 4. Results and analysis

### 4.1 Distribution characteristics of exposure risk at bus stops

**Fig 2:**
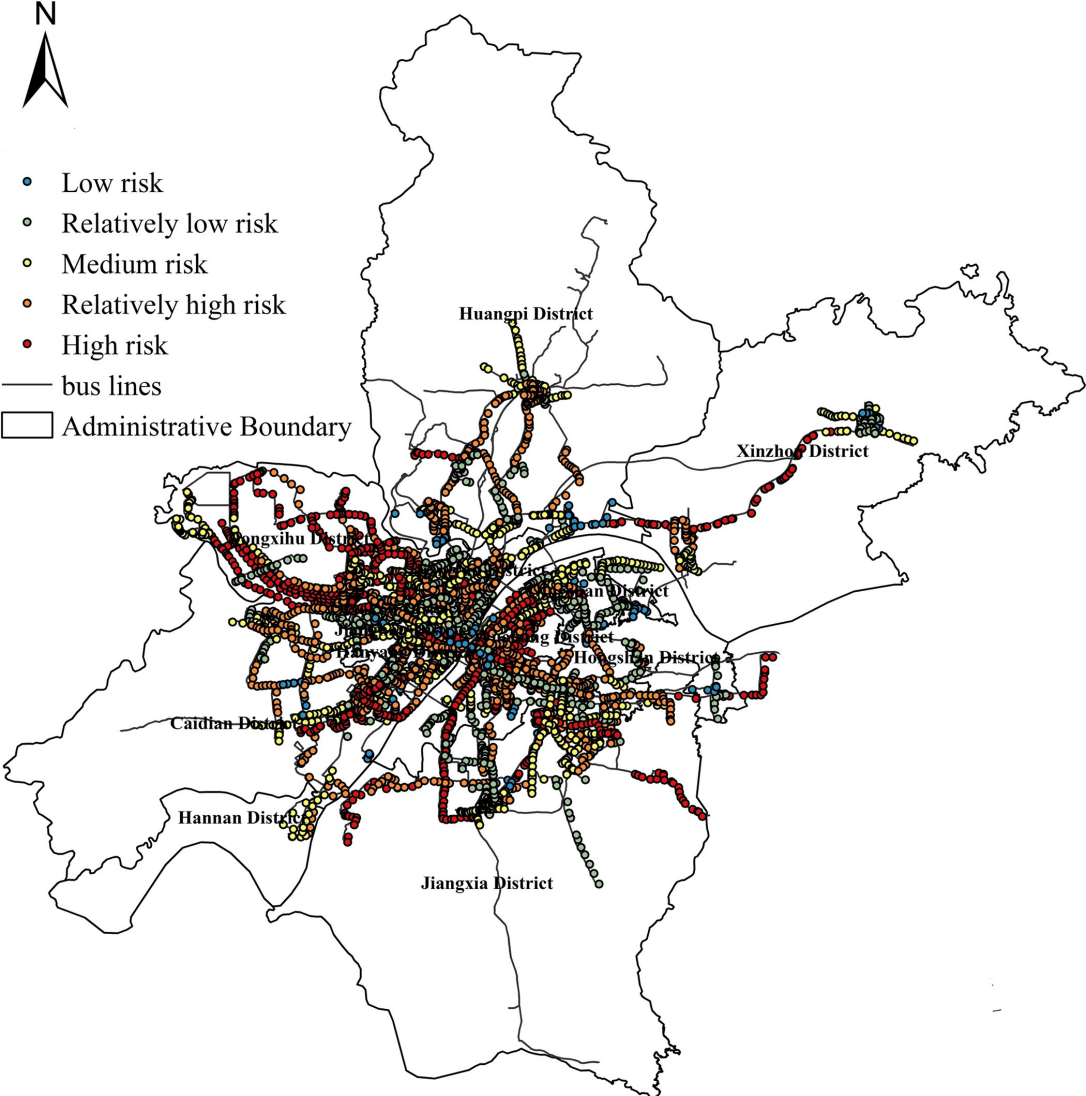
Exposure risk calculation results at bus stops.

**Fig 3:**
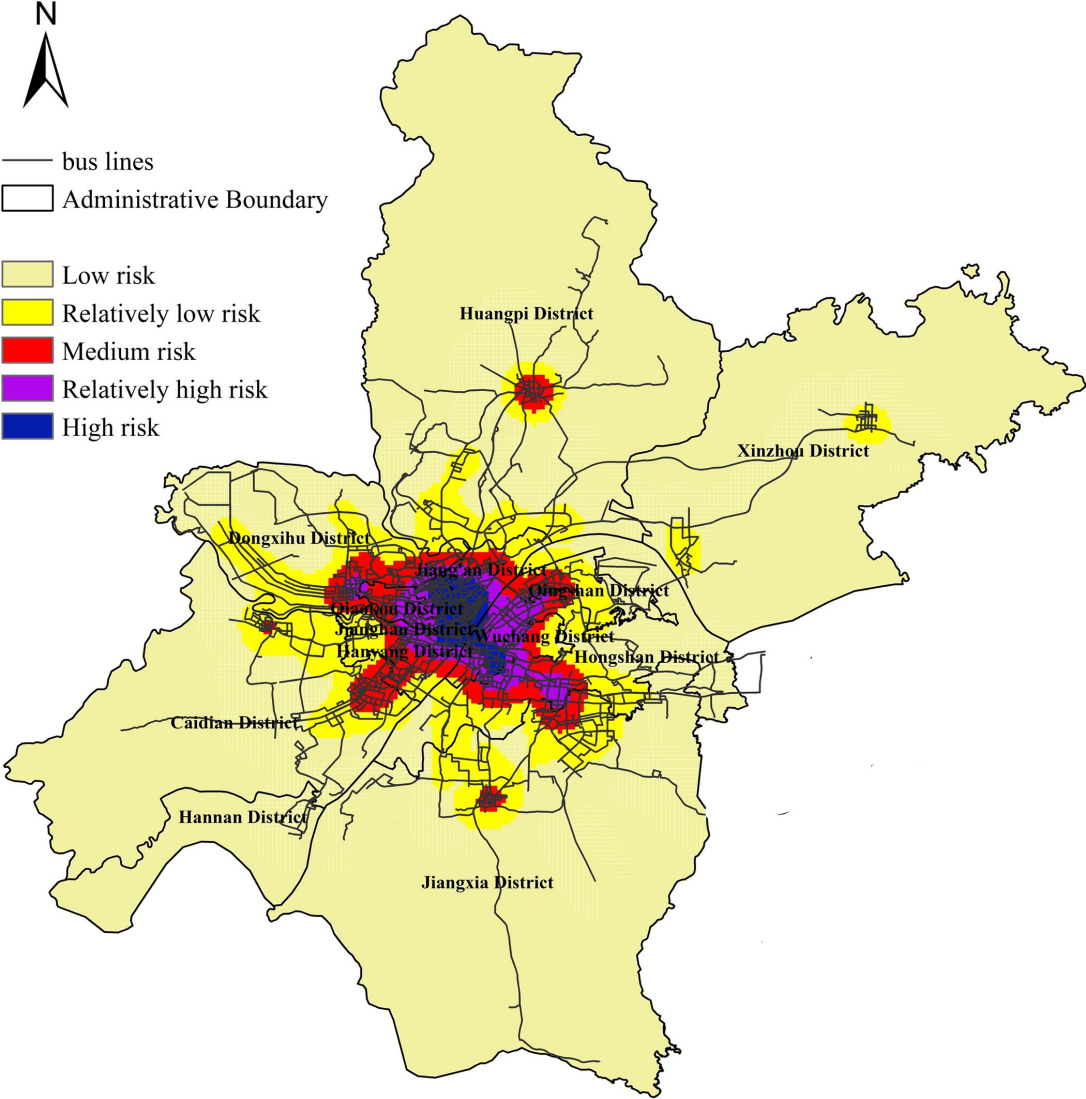
Spatial distribution of exposure risk at bus stops.

The exposure risk of all bus stops in Wuhan city is calculated. The classification of risk level is based on a clustering method commonly used in ArcGIS software: Natural Breaks method (Jenks). Its basic principle is that based on the inherent natural grouping in the data, the classification interval can be identified, the most appropriate grouping of similar values can be carried out, and the difference between various classes can be maximized. In this paper, the risk value calculated by the model is divided into five categories according to the Jenks method, namely high, relatively high, medium, relatively low and low. The results are as shown in Fig 2. From the perspective of spatial distribution, high risk and relatively high risk stops mainly rely on the main urban roads, such as Yanjiang Avenue, Zhongshan Avenue, Jiefang Avenue, etc., and the low risk stops are mainly concentrated on the city branch road. Further interpolation analysis shows that the overall exposure risk of Wuhan’s bus stops presents an obvious "multi center circle" structure. In other words, from the center circle layer to the outside, gradually decreasing, forming a pattern of "high in the South and low in the north, dense in the West and sparse in the East", specific spatial distribution results are as shown in Fig 3. The high risk sites are mainly concentrated in Wuchang District, Hanyang District, Qiaokou District, Jianghan District and other administrative regions. We can find out that the high-density land use development mode makes the exposure risk of bus stops appear obvious spatial agglomeration difference. It reflects the interaction between urban public transport exposure risk and land use. The exposure risk of bus stops is arranged in descending order, and polynomial function is used for fitting analysis. The result shows in S1 Appendix. The goodness of fit reaches 0.9855, and the fitting effect is good. It shows that there are a few stops in the whole network with high or low exposure risk, while most of the other stops have little difference in exposure risk.

The top 35% of high exposure risk stops are scattered in space, but they are mainly important transport hubs, tourist distribution centers and other important service facilities in cities, such as airport terminals, railway stops, stadiums, etc.; Similarly, the low exposure risk stops in the lower 30% are mainly village communities and neighborhood committees in the periphery of the core urban areas of each administrative region. The specific results are as shown in Table 1.

**Table 1:** Statistical analysis of exposure risks at different levels of bus stops.

Exposure risks of bus stops	High risk	Relatively high risk	Medium risk	Relatively low risk	Low risk
**Number**	1483	2440	3368	3100	618
**Percentage**	13.47%	22.16%	30.59%	28.16%	5.62%
**Typical bus Stops (Partly)**	Xin Rong Depot Xin Shi Gong Lu Qu High School Zhu Cheng depot Dong Shan Bus Station Gaoxin Avenue Railway Station	Jianshe Avenue science and Technology Museum Jiefang Avenue Tongji Hospital Dunkou Sports Center parking lot Jinshan Avenue Meeting Center Wuxi road Bus Station	103 Avenue detention center Huangling community Lingfeng road community Junjiang road Park Wugang Power Plant Huanshan road Military training base Pingjiang Avenue Power Plant	Huangpu Avenue Hospital Hanshi Avenue New countryside Jiangdi road Ecological park Linjiang Avenue Gongyuan road Zhanqian road Changqian market Weihu road Bus Station	Caidian Avenue Transportation Bureau Luo jiazui Bus Station Guoji middle Bus Station Minzhu road Decoration market Qingnian road City Museum

### 4.2 Distribution characteristics of exposure risk at epidemic sites

**Fig 4:**
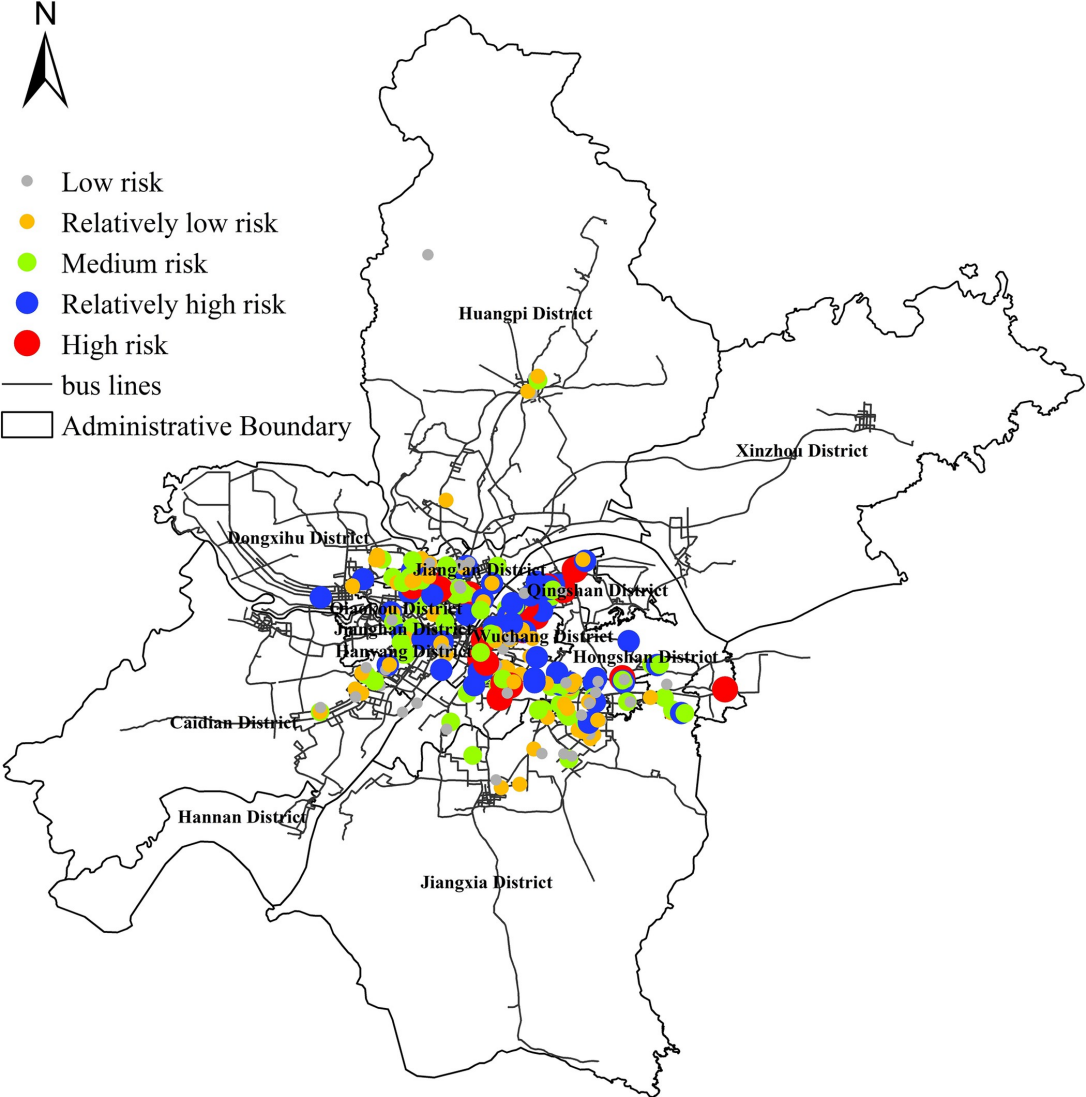
Spatial distribution of public transport exposure risk at epidemic sites.

In this section, the buffer zone of epidemic sites is taken as the analysis unit to measure the exposure risk of public transport at epidemic sites. It is also divided into five levels according to the natural breakpoint classification method. The specific results are as shown in Fig 4.

The results showed that the coverage of epidemic sites cover 4018 bus stops, accounting for about 36.5% of all urban public bus network stops, and 169 bus lines, accounting for about 39.9% of all bus lines in the area. The high-risk epidemic sites are mainly concentrated in the core area of Wuhan city center, and the low-risk epidemic sites are mainly distributed in the outer circle of the city. Further statistical analysis of the exposure risk at epidemic sites is carried out, and the public transport exposure risk at epidemic sites was arranged in descending order, and the overall distribution is fitted with polynomial function. The result shows in S1 Appendix. The goodness of fit R^2^ was 0.986, which shows that except for a few epidemic sites with high risk and low risk, the difference of exposure risk in most sites is not particularly significant. The distribution curve of exposure risk at epidemic sites is close to the overall distribution law of exposure risk at bus stops because under the influence of the public transport network, due to people’s mobile interaction behavior in the public transport network, the greater the exposure risk of bus stops, the more it can affect the exposure risk of epidemic sites. Therefore, the exposure risk distribution of epidemic sites will tend to be consistent with the exposure risk distribution of bus stops.

## 5. Conclusion and discussion

With the continuous spread of COVID-19 in Wuhan city, based on the multi-source data such as public transport network and epidemic information, this paper considers the two aspects of bus stops and epidemic sites, uses the basic principle of complex network, integrates ArcGIS spatial analysis tools such as the topological model of public transport network structure, the centrality model of public transport network and nuclear density analysis, the risk assessment model of public transport exposure is constructed. Based on the public transport exposure risk and its spatial distribution characteristics, this paper puts forward a reasonable and effective emergency management strategy for urban public transport, and demonstrates it through an example.

The results show that the overall spatial exposure risk of Wuhan city presents an obvious "multi center circle" structure at the level of bus stops. In other words, from the center circle layer to the outside, it forms a pattern of "high in the South and low in the north, dense in the West and sparse in the East". The high and relatively high risk stops mainly rely on the main urban roads, and the low and medium risk stops are mainly concentrated in the urban branches. The high and relatively high risk stops are mainly transport hubs, shopping malls and other sites, accounting for 35.63%. The medium and low-risk stops are mainly the villages and communities outside the core areas of each administrative region, accounting for 64.37%. On the other hand, at the scale of epidemic sites, the coverage covers 4018 bus stops in Wuhan, accounting for 36.5% of all bus stops, and 169 bus lines, accounting for 39.9% of all routes. High risk epidemic sites are mainly concentrated in the core areas within the jurisdiction of Wuhan City, and in the direction of urban outer circle diffusion, they are mainly distributed in the low and medium risk epidemic sites. The exposure wind of epidemic sites is mainly located in the core area of Wuhan city. The overall distribution of risk is similar to the overall distribution of urban public transport exposure risk at bus stops. In other words, a few sites are at high exposure risk, the risk value is significantly higher than other risk level sites, and the vast majority of sites are in medium and low exposure risk, and the overall difference is not obvious.

The public transport exposure risk caused by major public health emergency is a hot and difficult problem at present. Therefore, the management and control of urban public transport in the emergency stage has become a comprehensive and complex work. Especially for the super large, high-density and development-oriented city such as Wuhan, the daily travel of residents depends on the urban public transport system. The complete shutdown of the system in a large area will have a great impact on the social and economic operation of the city.

On the whole, in order to ensure the necessary traffic service function of the city and provide strong support for blocking the spread of the epidemic, it is necessary to assess the exposure risk of urban public transport under major public health emergency from different scales of the city, and obtain the exposure risk and its spatial distribution characteristics, so as to identify the bus stops and epidemic sites with high exposure risk. Finally, according to the difference of the risk level of public transport exposure, the hierarchical public transport control measures are formulated to achieve differentiation and accurate prevention and control, so as to provide theoretical basis and decision-making reference for the relevant departments to carry out risk management and formulate management and control policy, we put forward the following conclusions based on the existing research [27–29]:

- For high and relatively high exposure risk bus stops and epidemic sites. 1. We can select the regular bus lines involved in the outage to prevent the spread of COVID-19 in the city to the greatest extent. 2. We can divert public transport users to private transport modes, i.e. using walking, bicycles, cars and other means of transport. 3. For medical staff and public service personnel, it is necessary to coordinate the dispatching of buses to point" service, arrange special vehicles to pick up and send supermarket staff to and from work, set up emergency transport fleet to provide commuting support for medical institutions. 4. For commuters, it is necessary to customize the real name system of fixed crowds and fixed passenger capacity bus commuter line with each community as the starting point during rush hours.
- For bus stops and epidemic sites with medium exposure risk. 1. We can consider changing the route of some bus lines passing through the area, or not stopping at high-risk bus stops, so as to reduce the probability of virus infection in other low-risk stops and regions. 2. We can reserve the bus trunk lines connecting transport hubs, hospitals and covering the basic direction of the city, so as to ensure the basic travel and emergency travel needs of citizens 3. To reduce the departure frequency, transfer rate, adjust service time and limit the number of people on board, set up temporary lines according to the actual situation of passenger flow, such as large stop bus, inter district bus, etc.
- For bus stops and epidemic sites with low exposure risk. 1. We should disinfect buses, stops and other relevant equipment to prevent virus transmission to the greatest extent. 2. We can formulate flexible and staggered personalized travel routes. 3. We can take buses through declaration of health information, and check the temperature of passengers before boarding, so as to strictly control the crowd in and out. 4. We should encourage sharing bicycles and sharing vehicles, unmanned distribution and other non-contact transport services.

## Supporting information

S1 Appendix (DOCX)
