# Supplementary material for: Evaluation and prevention and control measures of urban public transport exposure risk under the influence of COVID-19—Taking Wuhan as an example
Source: PLoS One. 2022 Jun 6;17(6):e0267878. doi: 10.1371/journal.pone.0267878 (PMC9170111; doi:10.1371/journal.pone.0267878)
Supplement: S1 Appendix — (DOCX) [file pone.0267878.s001.docx]

**Appendix**

**Figure a：**

Polynomial fitting function of exposure risk value of bus stops


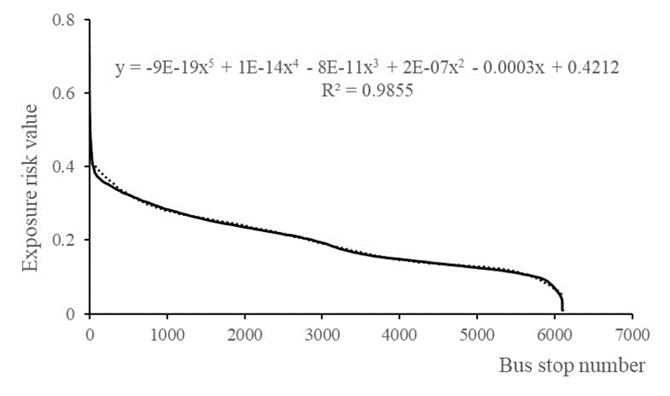


Figure a

**Figure b：**

Polynomial fitting function of exposure risk value of epidemic sites


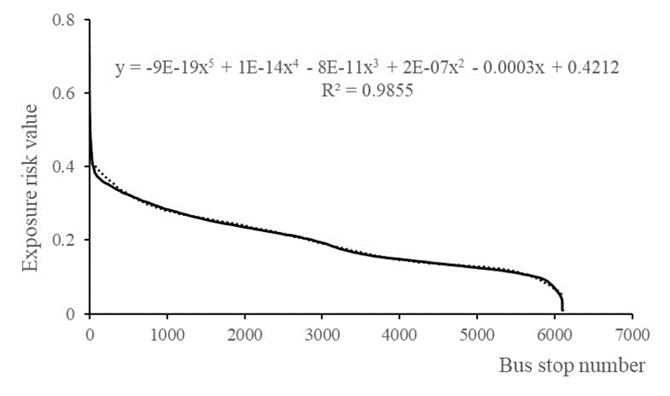


Figure b
